# ATP13A2 is involved in intracellular polyamine transport in lung epithelial cells

**DOI:** 10.1002/2211-5463.70158

**Published:** 2025-11-18

**Authors:** Yuta Hatori, Kohei Kawabata, Takanori Kubo, Takeo Kitazawa, Sae Kanai, Madoka Iwashita, Ami Hayashi, Hiroyuki Nishi, Mikihisa Takano

**Affiliations:** ^1^ Faculty of Pharmacy Yasuda Women's University Hiroshima Japan

**Keywords:** ATP13A2, lung, polyamine

## Abstract

Polyamines are present in all living cells and are implicated in various crucial cellular processes such as proliferation, apoptosis and autophagy. In contrast, excess amounts of polyamines can be toxic to cells. ATP13A2 was recently identified as a mammalian polyamine transporter in neuronal cells. In this study, we attempted to characterize the function of ATP13A2 in cultured human lung epithelial cells. The data demonstrated that ATP13A2 is endogenously expressed in mouse lungs, and in cultured lung epithelial cells, the expression level of ATP13A2 drastically changes over culture time and peaks in the logarithmic phase during the proliferation curve. ATPase activity in ATP13A2‐enriched microsomes from lung cells showed polyamine dependence, as previously reported for other cell types and species. Overexpression of ATP13A2 caused a moderate increase in total cellular polyamine content, whereas ATP13A2 knockdown resulted in a decrease in cellular polyamine levels. These findings provide novel information regarding the cellular function of ATP13A2 in lungs and contribute to our understanding of cellular polyamine transport systems.

## Graphical abstract

Spermidine transport in lung epithelial cells involves the polyamine transporter ATP13A2. Cell proliferation is associated with the upregulation of ATP13A2.

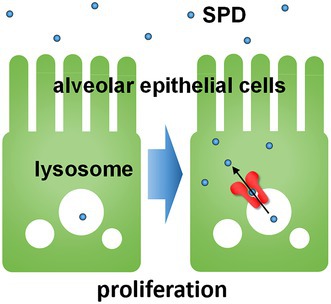

**Abbreviations**
DTT: dithiothreitol
MOI: multiplicity of infection
OG: oregon green
PUT: putrescine
SPD: spermidine
SPM: spermine
TCEP: tris(2‐carboxyethyl)phosphine
TMR: tetramethylrhodamine

Polyamines are low‐molecular weight aliphatic amines that are biosynthesized in all kingdoms of life [1]. Naturally synthesized polyamines include putrescine (PUT; NH_2_ (CH_2_)_4_ NH_2_), spermidine (SPD; NH_2_ (CH_2_)_4_ NH (CH_2_)_3_ NH_2_) and spermine (SPM; NH_2_ (CH_2_)_3_ NH (CH_2_)_4_ NH (CH_2_)_3_ NH_2_). Polyamines are primarily present as cations at physiological pH and are capable of electrostatic interactions with anionic biomolecules, such as nucleic acids and lipid bilayers. In addition, their amino groups serve as radical scavengers and exert cytoprotective effects against oxidative insults [2]. Owing to these unique biochemical properties, polyamines are implicated in several fundamental cellular processes, such as proliferation [3], apoptosis [3] and autophagy [4]. More recently, polyamines have been found to play broader roles in antibody production [5] and the circadian clock [6]. Although polyamines play pivotal physiological roles, excess polyamines can exert cellular toxicity. In rats, inhalation of SPD causes necrotic loss of club cells and Type II pneumocytes, resulting in irreversible alveolar injury and subchronic pneumonitis [7]. Cultured cells exposed to high concentrations of SPD and/or SPM show reduced growth rate [8]. These cytotoxic effects of polyamine are mainly attributed to reactive catabolites of polyamine such as acrolein (acrylic aldehyde) [5], a potent electrophilic compound with an IC50 of 60 μm [9]. In bacteria, the accumulation of SPD induces mRNA deformation and hinders translation [10]. Thus, abnormal polyamine levels, both too low and too high, can be detrimental to cells, indicating the necessity of fine‐tuned systems for controlling cellular polyamine dynamics.

Although the physiological importance of polyamine regulation is ubiquitous in the human body, the lungs are considered to possess a particularly active polyamine uptake system [11]. In this study, we characterized the function of the recently identified mammalian polyamine transporter, ATP13A2 [12, 13, 14, 15], in cultured lung epithelial cells. For biochemical characterization, we prepared recombinant adenoviruses and overexpressed ATP13A2 in cultured lung cells. The findings and methods described in this study provide novel information regarding the cellular function of ATP13A2 in lungs and contribute to our understanding of the polyamine transport system.

## Materials and methods

### Antibodies

The antibodies were purchased from the manufacturers listed in Data S1.

### Tissue samples

Preparation of mouse tissue lysate was carried out in strict accordance with the recommendations in the Guide for the Care and Use of Laboratory Animals of the National Institutes of Health. The protocol was approved by the Committee on the Ethics of Animal Experiments of Yasuda Women's University (protocol number: BB2306).

Six‐week‐old ICR male and female mice were purchased from CLEA Japan, Inc. After sacrificing the mice under euthanasia by isoflurane, the brain, small intestine, colon, liver, pancreas, lung, heart, kidney and spleen were quickly removed and stored. Preparation of mouse tissues was carried out in strict accordance with the recommendations in the Guide for the Care and Use of Laboratory Animals of the National Institutes of Health. The protocol was approved by the Committee on the Ethics of Animal Experiments of Yasuda Women's University (protocol number: BB2306).

The tissues were cut using a scalpel, ground in liquid nitrogen and homogenized in ice‐cold lysis buffer 50 mm HEPES, pH 7.5, 150 mm NaCl, 10% glycerol and Protease Inhibitor Cocktail for Use with Mammalian Cell and Tissue Extracts (Nakalai). Crude membrane was isolated by the procedure described in the Section 2.5. membrane preparation.

### Cell culture

HEK293A cells were obtained from Thermo Fisher Scientific (R705‐07) and maintained in Dulbecco's Modified Eagle Medium (DMEM) supplemented with 10% FBS, 50 U·mL^−1^ penicillin and 50 μg·mL^−1^ streptomycin (Thermo Fisher Scientific, Waltham, MA, USA). Plasmids were transfected using Lipofectamine 3000 and Opti‐MEM (Thermo Fisher Scientific). NCI‐H441 cells were obtained from American Type Culture Collection (HTB‐174; ATCC, Manassas, VA, USA) and maintained as previously reported [16, 17]. Briefly, the cell line was cultured in RPMI‐1640 medium supplemented with 5% FBS, 1 mm sodium pyruvate, 100 U·mL^−1^ penicillin and 100 μg·mL^−1^ streptomycin (Thermo Fisher Scientific). Two days after seeding, the medium was replaced with complete medium containing insulin‐transferrin‐selenium (ITS). Culture medium was replenished every 2 days. All cells were cultured at 37 °C in a humidified atmosphere of 5% CO_2_.

### Construction of recombinant adenovirus

Adenovirus carrying human ATP13A2 cDNA was constructed using ViraPower Adenoviral Expression System (K4930‐00; Thermo Fisher Scientific) according to the manufacturer's instructions. Plasmid pHTC/ATP13A2‐Halo was used as the original source of the cDNA [18]. The coding region containing human ATP13A2, TEV recognition site, and Halo tag was amplified using the primer set F_pENTR_13A2HaloHis (5′‐CACCATGAGCGCAGACAGCAGCCCTCTC‐3′) and R_pENTR_13A2HaloHis (5′‐TTAatgatgatgatgatgatgACCGCCACCGGAAATCTCCAGAGTAGAC‐3′ where lower case letters represent the 6 × His tag sequence), resulting in ATP13A2‐Halo sequence fused with the C‐terminal 6 × His tag. The amplified DNA fragment was subcloned into pENTR/D‐TOPO vector (K2400‐00; Thermo Fisher Scientific), which was subsequently reacted with pAd/CMV/V5‐DEST Gateway Vector (V493‐20; Thermo Fisher Scientific) for *in vitro* homologous recombination. The adenoviral clone DNA was linearized by PacI digestion and transfected into HEK293A cells using Lipofectamine 3000. The cells were maintained for 1 week until recombinant virus was successfully packaged, and infection‐associated cytopathic effect was observed. Infected cells were collected, and viruses were collected by freeze–thaw cycle, generating P1 virus suspension. For clonal selection, HEK293A cells were infected with P1 virus at 10^−3^ MOI and layered with complete medium supplemented with 2% agarose. Infection plaques were separately picked up using Pasteur pipets and virus clones were recovered by freeze–thaw cycle. Each virus clone was separately amplified and tested for overexpression of recombinant ATP13A2. Among 12 clones, ATP13A2 expression was successfully detected for all the clones and the one producing the best result was selected for conducting further experiments. For mock conditions, adenovirus carrying LacZ gene was also prepared in the same way.

### Microsome preparation

H441 cells were infected with either mock virus (Ad/LacZ) or ATP13A2 virus at 10 MOI and further incubated for 48 h. Cells were then harvested and washed with ice‐cold PBS twice. Washed cells were resuspended in 10 mm Tris, pH8.0 supplemented with protease inhibitor cocktail (04080‐11; Nakalai Tesque) and processed by Dounce glass homogenizer. Mouse tissues were also processed using the same procedures. Tris pH8.0, glycerol and NaCl were further added to the sample to obtain the final composition of 25 mm Tris–HCl, pH8.0, 10% glycerol and 150 mm NaCl. The cell suspension was centrifuged for 30 min at 5000 ***g*** and the sedimented debris was removed. Cleared lysate was centrifuged for 4 h at 45 000 ***g*** and pelleted microsome was resuspended in the buffer of the same composition. Protein concentration was determined by Pierce BCA Protein Assay—Reducing Agent Compatible (23250; Thermo Fisher Scientific). Typically, protein concentration was above 10 mg·mL^−1^. Microsome preparation was stored in a −80 °C deep freezer and kept no longer than 2 weeks.

### ATPase activity assay

Microsome sample was diluted in a buffer (ATPase assay medium) containing 20 mm Tris–HCl, pH8.0, 10% glycerol, 1 mm DTT, 5 mm MgCl_2_ and 2 mm ATP. The reaction was initiated by adding MgCl_2_. The reaction tubes were sealed and incubated in a water bath at 37 °C for 30 min. ATP hydrolysis was terminated by mixing with 1% ammonium molybdate solution containing 1 N HCl (AM solution). Addition of AM solution is a part of the protocol to quantitate inorganic phosphate documented by Lanzetta *et al*. [19]. ATPase activity was typically measured in the absence and presence of ligands such as 2 mm SPD and ligand‐dependent activity was obtained by subtraction. The data of polyamine dependence was fit to the Hill equation using Prism (GraphPad Software).

### Confocal microscopy

Cells were seeded to a cover glass chamber slide (5232‐008; Iwaki AGC, Shizuoka, Japan). The glass surface was coated with type‐I collagen (354236; Corning, Corning, NY USA). Chamber samples were placed on a temperature‐ and atmosphere‐controlled stage of the confocal microscope FV1000 (Olympus, Japan). Images were analysed and processed using Image J [20] with the Fiji package [21].

### Polyamine quantification

Cells were seeded on a 6‐well plate at 50% density. The next day, cells were infected with the recombinant ATP13A2 adenoviruses at 5 MOI and further cultured for 1 day, followed by 24 h of incubation with either 1 mm PUT, SPD, SPM or basal condition. In some conditions, cells were incubated with 10 μm n‐n′‐dimethyl formamide (DMFO) for 1 day which inhibits ornithine synthase and thereby suppresses biosynthesis of endogenous polyamines. Cells were then washed with PBS twice, and lysed by adding 1 mL of 0.1 N HCl. Cells were further lysed by ultrasonication and debris was removed by centrifugation at 3000 ***g*** for 10 min. Cleared cell lysates were subjected to BCA protein assay and polyamine quantification. Derivatization, extraction and quantification of polyamines were carried out essentially as described by a previous study [22]. In brief, cell lysate (10 μL), internal standard (IS, 1,6‐diaminohexane, 12.5 ng) in 0.1 M HCl (10 μL), 0.1 m Borax (pH 9.3, 30 μL) and 4‐(*N,N*‐dimethylaminosulfonyl)‐7‐fluoro‐2,1,3‐benzoxadiazole (DBD‐F, 200 μg) in acetonitrile (50 μL) were added and the mixture was heated at 60 °C for 30 min. The reaction mixture was diluted with water (200 μL), and derivatized polyamine and IS were extracted by the addition of ethyl acetate (200 μL). An ethyl acetate phase was transferred to another tube and the solvent was removed. The residue was dissolved in the mobile phase, and an aliquot of the solution was subjected into liquid chromatography/electrospray‐ionization tandem mass spectrometry (LC/ESI‐MS/MS) system (LCMS‐8040; Shimadzu Corp., Kyoto, Japan). The ratio of peak area for IS was utilized for the calculation of PUT, SPD and SPM contents based on the values obtained from calibration curves.

### SDS PAGE and western blotting

Proteins were typically separated using 7.5%–acrylamide SDS gels. Before electrophoresis, each protein sample was combined with one third volume of 4 × Laemmli sample buffer solution (198‐13282; FUJIFILM Wako Pure Chemical Corporation, Osaka, Japan) containing 2 mm tris(2‐carboxyethyl)phosphine (TCEP). To observe protein bands, gels were stained with QC Colloidal Coomassie Stain (1610803; Bio‐Rad, Hercules, CA USA). For detection of Halo‐tagged protein, 25 μg of microsomal protein was reacted with 10 μm TMR Halo‐ligand (Promega, Madison, WI, USA) for 20 min at room temperature and then subjected to electrophoresis.

For western blotting, separated proteins were transferred to PVDF membranes. Blocking was performed using blocking one reagent (03953‐95; Nakalai Tesque) and the membrane was incubated with primary antibodies at 4 °C overnight, followed by the reaction with secondary antibodies at room temperature for 2 h. Chemiluminescence was detected using ECL Prime (Cytiva, RPN2232) and LAS‐4000 lumino image analyser (Fujifilm, Tokyo, Japan). Densitometric analyses were performed using Image J with the Fiji package.

### Real time quantitative PCR (RT‐qPCR)

Total RNA was extracted using the RNeasy Mini Kit (Qiagen, Venlo, The Netherlands, UK) and quantified using NanoDrop One^c^ (Thermo Fisher Scientific). The primers used for target mRNAs were as follows: ATP13A1 forward primer, 5′‐CGCTGACCAAAGATGAGAAA‐3′; ATP13A1 reverse primer, 5′‐TTCTCATACGAGGCAAGCAC‐3′; ATP13A2 forward primer, 5′‐AGGACCAAATGGTGAGGAAG‐3′; ATP13A2 reverse primer, 5′‐GGAGATGGAGGAAATGAGGA‐3′; ATP13A3 forward primer, 5′‐GAGTGCTCTCTGGTGATCCA‐3′; ATP13A3 reverse primer, 5′‐AGTTGTTTGGGAGGACGAAC‐3′; ATP13A4 forward primer, 5′‐ CCTGGTGCCTGGAGATTTAT‐3′; ATP13A4 reverse primer, 5′‐CCATCTTGGGTAACGGAGTT‐3′; GAPDH forward primer, 5′‐GCACCGTCAAGGCTGAGAAC‐3′; GAPDH reverse primer, 5′‐TGGTGAAGACGCCAGTGGA‐3′. RT‐qPCR was performed using a Thermal Cycler Dice Real‐Time System III (Takara Bio, Shiga, Japan) with a Luna Universal one‐step qPCR kit (New England BioLabs, Ipswich, MA, USA), according to the manufacturer's protocol. Relative quantification was performed by preparing standard curves. GAPDH was used as an endogenous control.

### Knockdown of ATP13A2 and ATP13A3

H441 cells were spread on a 24‐well plate at a cell density of 1.0 × 10^5^ cells per well. One day after cell seeding, cells were transfected with siRNAs; 50 nm siATP13A2 (Dharmacon, Lafayette, CO, USA; M‐008601‐01‐0005), 50 nm siATP13A3 (Dharmacon M‐008131‐01‐0005), 50 nm siATP13A2 plus 50 nm siATP13A3 or 50 nm siControl (non‐targeted siRNA). Transfection was performed using RNAiMAX (Thermo Fisher Scientific) as per the manufacturer's instructions. Culture medium was replenished 1 day after transfection and further incubated for 1 day, followed by qPCR analysis or polyamine transport assay.

### Statistical analysis

Data points with error bars in each plot represent mean values and standard deviations obtained from three independently prepared samples where indicated. Significance was assessed by Student's or Welch's *t*‐tests using Prism (GraphPad Software). *P* values of < 0.05 were regarded as statistically significant.

## Results

Mutations in ATP13A2 have been associated with Kufor‐Rakeb syndrome [23] and its molecular function has been investigated primarily in neuroblastoma cells, such as SH‐SY5Y or more versatile cell lines, including HeLa, HEK293, CHO and COS‐1 cells. Although the function of ATP13A2 is important in the brain, its possible systemic role has not been well characterized. Based on the actively operating polyamine transport system [11] and our original pharmacokinetic interest in the lung epithelium [24, 25, 26], we attempted to define the potential role of ATP13A2 in the lung.

**Fig. 1:**
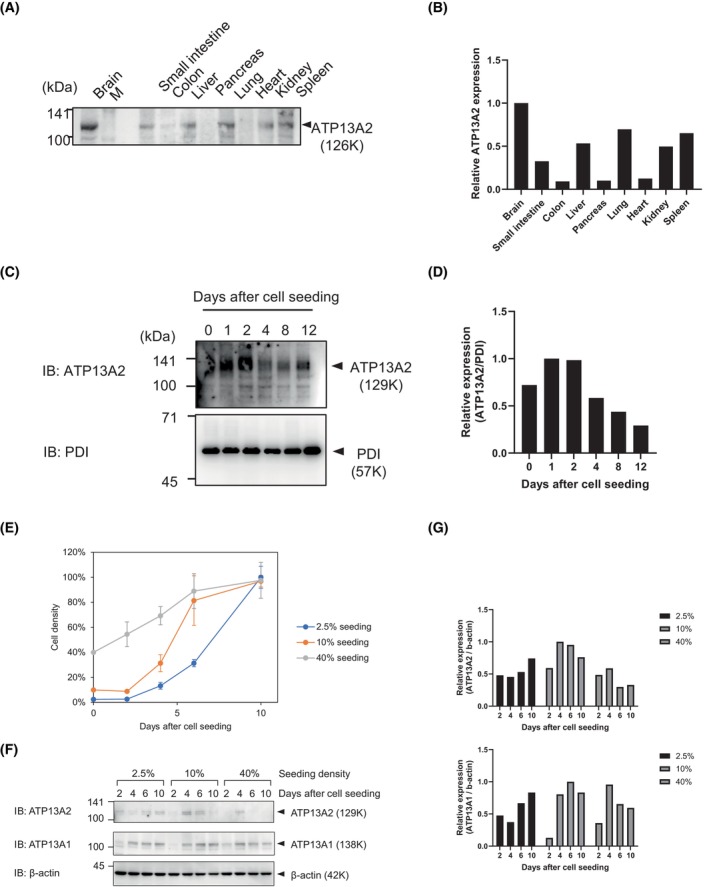
Endogenous expression of ATP13A2 in the lung and cultured Type II alveolar epithelial cells (NCI‐H441). (A) Detection of ATP13A2 in mouse organs. Microsome was isolated from distinct organs and tested for ATP13A2 expression by western blotting. Brain microsome was used as positive control. Equal amount of protein (20 μg) was loaded to each lane. M signifies a molecular weight marker. The same set of protein samples were subjected to SDS‐PAGE and stained by Coomassie Brilliant Blue for densitometric quantification of total loaded protein (Data S2). (B) ATP13A2 bands were quantified and normalized by total loaded protein amounts (Data S2). (C) Detection of endogenous ATP13A2 in human alveolar type II epithelial cells (NCI‐H441). Cells were seeded at 20% density and harvested at different time points from 0 to 12 days after cell seeding. Culture medium was replenished every 2 days. Microsome fractions were analysed for ATP13A2 expression. Equal amount of protein (20 μg) was loaded to each lane and polypeptide disulfide isomerase (PDI) was used as loading control. (D) ATP13A2 bands in *C* were quantified and normalized to PDI. (E) Growth curves of H441 cells seeded at various densities (2.5, 10 or 40%). Live cells were counted at different time points. Trypan blue was used for staining of dead cells. Typically, the ratio of live/dead cells was over 95%. Data are presented as mean ± S.D obtained from three independently prepared samples. (F) Detection of endogenous ATP13A2 in H441 cells seeded at various densities (2.5, 10 or 40%) at different time points. Microsome fractions were analysed for ATP13A2 expression. Equal amount of protein (20 μg) was loaded to each lane and β‐actin was used as loading control. (G) ATP13A2 and ATP13A1 bands in *F* were quantified and normalized to β‐actin.

First, we compared ATP13A2 expression levels in various mouse tissues (Fig. 1A,B). Microsomal fractions were isolated to observe ATP13A2 protein bands. The expression levels were normalized to the total loaded protein visualized using a Coomassie brilliant blue‐stained gel (Data S2). As demonstrated in a previous study [23], the major site of ATP13A2 expression is the brain. When comparing non‐neuronal tissues, prominent tissue specificity was observed, indicating significant expression in the lungs and liver. Similarly, in cultured human lung cells (H441), endogenous expression of ATP13A2 was demonstrated (Fig. 1C,D). Interestingly, ATP13A2 expression was altered in a time‐dependent manner. After cell seeding, the expression peaked on day 2 and gradually decreased thereafter. Accordingly, the mRNA levels peaked on Day 1 and declined over time (Data S3), suggesting that ATP13A2 expression changes with culture time under transcriptional control. We considered two alternative possibilities: (1) ATP13A2 expression could change with the proliferative stage or (2) it could depend on the absolute time after cell seeding. If possibility (1) is true, the peak time point may change by modifying the time course of cell proliferation. To distinguish between these possibilities, we seeded cells at different densities (2.5%, 10%, and 40%) and observed density‐dependent shifts in the growth curve (Fig. 1E). In these three different time courses, ATP13A2 expression peaked on Days 10, 4 and 4, which correlated with the logarithmic periods within each growth curve (Fig. 1F,G). This correlation was unique to ATP13A2 and was not observed for ATP13A1, one of the other P5‐ATPases. Thus, ATP13A2 expression depends on the proliferative phase rather than the absolute time after cell seeding.

**Fig. 2:**
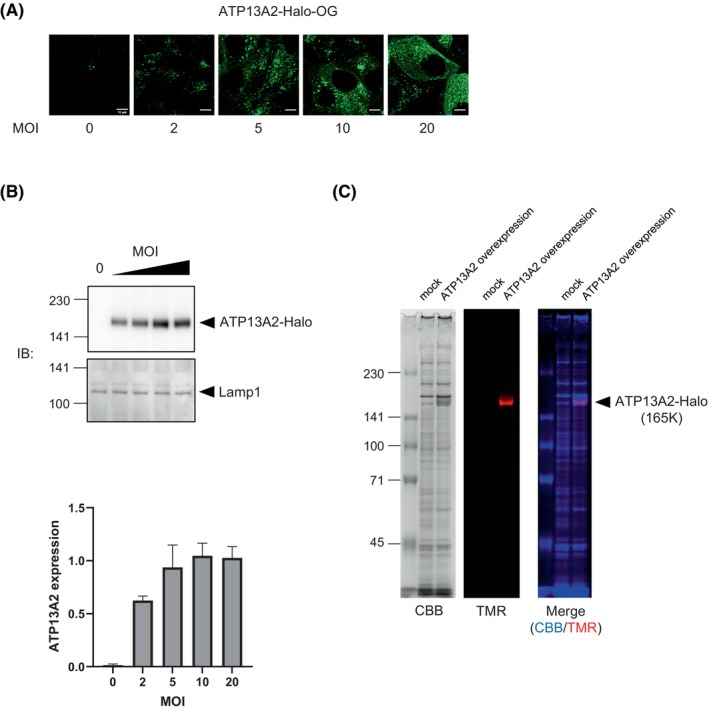
Adenovirus‐mediated overexpression of recombinant human ATP13A2 in H441 cells. Halo‐6 × His tag was C‐terminally fused with human ATP13A2 (ATP13A2‐Halo) and the cDNA was transferred into the adenovirus vector. Adenovirus was packaged and amplified in HEK293A cells. Cloned adenovirus was used for all the experiments. (A) Live cell images of H441 cells overexpressing ATP13A2‐Halo. H441 cells were infected with ATP13A2 adenovirus at different multiplicities of infection (MOIs). ATP13A2‐Halo was labeled with the fluorescent probe Oregon Green (OG) Halo‐ligand. Scale bar, 10 μm. (B) Detection of ATP13A2 in infected H441 cells (MOI 0, 2, 5, 10, and 20). Microsome fractions were analysed for ATP13A2 expression. An equal amount of protein (1 μg) was loaded to each lane. Lamp1 was analysed as an internal standard. Band intensities were quantified based on the results of multiple experiments (*n* = 4). Bars represent standard deviations. (C) Microsome fractions from H441 cells infected with mock (LacZ) and ATP13A2 adenovirus were analysed by SDS‐PAGE. ATP13A2‐Halo was specifically labeled *ex vivo* with tris(2‐carboxyethyl)phosphine (TMR)‐ligand. In the merged image, CBB and TMR bands were pseudocolored in blue and red, respectively. In this color arrangement, merged signals exhibit a purple color.

ATP13A2 has recently been identified as a novel polyamine transporter in cultured neuronal cells [15]. To elucidate the molecular function of ATP13A2 in the lungs, we characterized the biochemical properties of ATP13A2 overexpressed in H441 cells. Protein samples were prepared using an adenoviral vector system that allowed efficient and robust gene delivery to lung cells. For protein detection and isolation, a Halo tag was fused to the C‐terminus of human ATP13A2 (ATP13A2‐Halo). A recombinant adenovirus carrying ATP13A2‐Halo cDNA was generated by *in vitro* DNA recombination and packaging in HEK293A cells and further cloned by plaque isolation. ATP13A2‐Halo was successfully overexpressed in H441 cells without any cytopathic effects at an MOI range of up to 20 (Fig. 2A). ATP13A2‐Halo was mainly localized to the lysosomes (Data S4), as previously observed for endogenous proteins [27]. Western blotting detected only the full‐length ATP13A2‐Halo (Fig. 2B). For biochemical characterization, the microsome fraction was isolated using differential two‐step centrifugation. On the CBB‐stained gel, a protein band matching the theoretical mass of ATP13A2‐Halo (165 K) was observed (Fig. 2C).

**Fig. 3:**
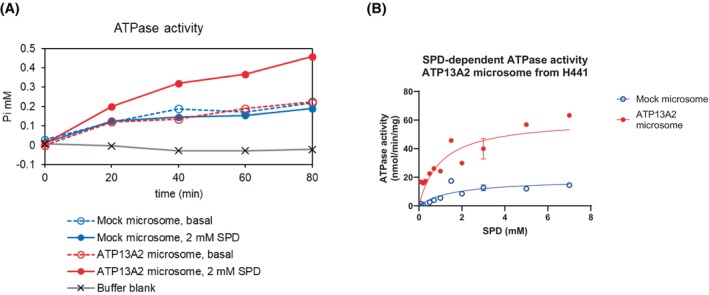
Polyamine‐dependent ATPase activity in microsome fraction isolated from H441 cells infected with ATP13A2 adenovirus. (A) ATPase activity was assessed by determining the amount of liberated inorganic phosphate (Pi). Microsome was incubated at 37 °C for up to 80 min in a buffer containing 20 mm Tris–HCl, pH8.0, 10% glycerol, 1 mm DTT, 5 mm MgCl2 and 2 mm ATP. Assay was performed in the absence (basal) and presence of 2 mm spermidine (SPD). Background was subtracted from each value. (B) Polyamine‐dependence of ATPase activity in mock and ATP13A2 microsomes. The assay condition was the same as used in *A* except for the reaction time (30 min). The plot for ATP13A2 microsome was fit to the Hill equation with the Hill coefficient *n* = 1 and *K*_d_ 1.00 mm. The plot for mock microsome was fit to the Hill equation with the Hill coefficient *n* = 1 and *K*_d_ 1.63 mm. Data points for 3 mm SPD are presented as mean ± S.D obtained from three independently prepared samples.

Microsomes prepared from ATP13A2‐overexpressing H441 cells were subjected to biochemical assays. Previous studies have demonstrated that ATP13A2 functions as a polyamine pump [12, 13, 14, 15]. Accordingly, we assayed the microsome preparations for SPD‐stimulated ATPase activity. ATP13A2‐containing microsomes showed time‐dependent ATP hydrolysis when stimulated by 2 mm SPD (Fig. 3A). Notably, the crude membrane fraction contained multiple endogenous ATPases. Indeed, mock microsomes exhibited significant ATPase activity that SPD did not stimulate. ATP13A2 microsomes showed similar levels of SPD‐independent components, which may represent endogenous microsomal ATPases rather than overexpressed ATP13A2. Polyamine dependence of ATP13A2 was fitted to the Hill equation with Hill coefficient *n* = 1 and *K*_d_ 1.00 mm (Fig. 3B). This substrate affinity is comparable to that of Na^+^/K^+^‐ATPase (K^+^, 0.91 mm [28]) and lower than that of Ca^2+^‐ATPase (0.26 μm [29]) and Cu^+^‐ATPase (2.1 μm [30]). The relatively low ligand affinity of ATP13A2, although not surprising based on the previously published results [15, 31], is a prominent feature that potentially reflects a high concentration range of intracellular polyamines (0.458 mm SPM and 0.175 mm SPD in the guinea pig heart [32]).

**Fig. 4:**
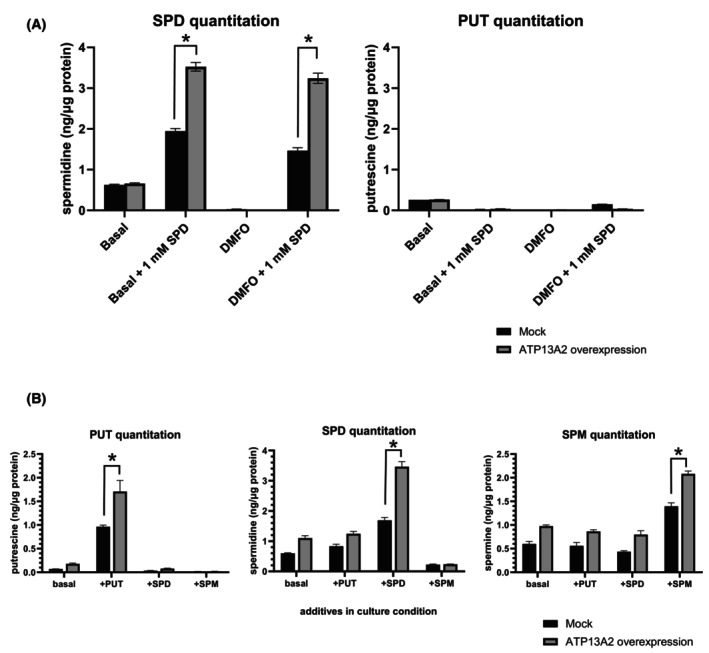
The effect of overexpression of ATP13A2 on cellular polyamine levels. (A) Intracellular SPD was quantified by mass spectrometry. The cells were incubated in the absence (Basal) or presence of 1 mm SPD. For comparison, putrescine (PUT) levels were also quantitated. Where indicated, cells were preincubated for 24 h with 10 μm n‐n′‐dimethyl formamide (DMFO), an inhibitor of polyamine biosynthesis. Data are presented as mean ± S.D (*n* = 3). Statistical analysis was performed using Student's *t* test: *P < 0.05. (B) H441 cells were incubated for 1 day in the presence of either 1 mm PUT, 1 mm SPD, 1 mm spermine (SPM) or basal condition, followed by polyamine quantification by mass spectrometry. Data are presented as mean ± S.D (*n* = 3). Statistical analysis was performed using Student's *t* test: *P < 0.05.

Next, we assessed the effects of ATP13A2 overexpression on cellular SPD levels. It was previously reported that ATP13A2 overexpression significantly increased the polyamine quantity within cells [12, 13, 14, 15], hence identifying this protein as a lysosomal efflux pump, which transports polyamines from the lumen into the cytosol across the lysosome membrane. We compared SPD quantity between mock and ATP13A2‐overexpressing cells using LC/ESI‐MS/MS analysis. Cells treated with an inhibitor of polyamine biosynthesis (n‐n′‐dimethyl formamide, DMFO) were used to deprive endogenously synthesized polyamines, demonstrating a clear decline in cellular SPD level. Unlike previous studies in other cell types and organisms, ATP13A2 overexpression alone failed to produce an evident increase in cellular SPD levels (Fig. 4A). Considering that cellular polyamine levels may be maintained by the equilibrium of synthesis/catabolism and influx/efflux, the effect of ATP13A2 overexpression could be masked by other components. Supporting this possibility, in the presence of exogenous SPD, overexpression of ATP13A2 resulted in a significant increase in cellular SPD compared to that in the mock condition. These data establish that ATP13A2 is involved in increasing the total level of intracellular SPD by facilitating the incorporation of extracellular SPD in H441 cells. We further tested the incorporation of other major polyamines, that is, PUT, SPM and SPD. Overexpression of ATP13A2 increased the incorporation of SPD, PUT and SPM (Fig. 4B), suggesting that ATP13A2 is involved in the incorporation of these three major polyamines.

**Fig. 5:**
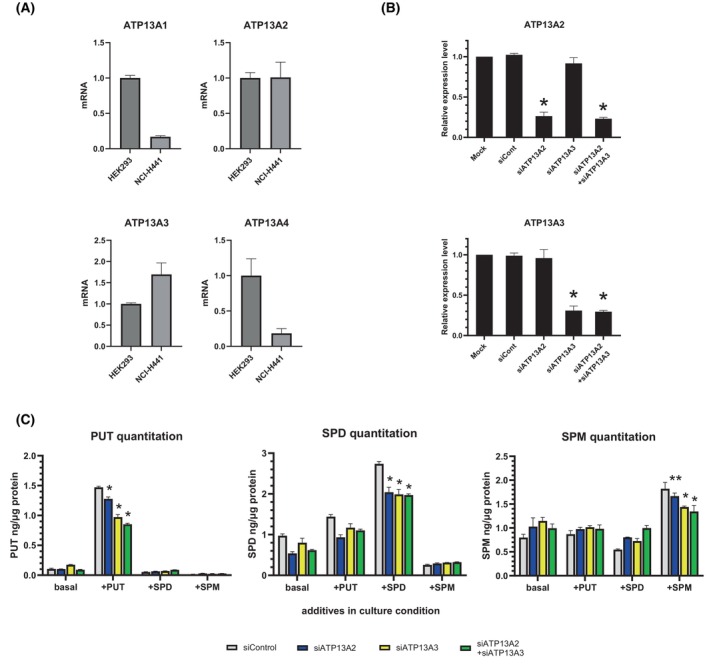
Effects of ATP13A2 and ATP13A3 knockdown on cellular polyamine levels. (A) Endogenous mRNA levels of P5‐ATPases in H441 cells. The values are relatively compared to HEK293, which was previously reported to be positive for ATP13A2 expression. Data are normalized to the value for HEK293 and presented as mean ± S.D (*n* = 3). (B) siRNA‐mediated knockdown of ATP13A2 and ATP13A3 in H441 cells. Cells were treated with either ATP13A2‐ or ATP13A3‐targeted siRNA or both. Data are normalized to values for Mock and presented as mean ± S.D (*n* = 3). Statistical analysis was performed using Student's *t* test: *P < 0.05 compared to the value for Mock. (C) H441 cells were first treated with siRNAs and then incubated for 1 day in the presence of either 1 mm PUT, 1 mm SPD, 1 mm SPM or basal condition, followed by polyamine quantification by mass spectrometry. Data are presented as mean ± S.D (*n* = 3). Statistical analysis was performed using Student's *t* test: *P < 0.05 compared to the value for Mock; **P < 0.1 compared to the value for Mock.

We explored the possible involvement of other P5B‐ATPases in polyamine handling in H441 cells. The mRNA levels of ATP13A2, ATP13A3 and ATP13A4 in H441 cells were quantified using the cell line HEK293 as reference because it was previously shown to be ATP13A2‐positive [33]. Based on the relative mRNA levels, ATP13A3 was potentially expressed in this cell line (Fig. 5A). Accordingly, we performed ATP13A2‐ and ATP13A3‐knockdown experiments to determine their contributions to cellular polyamine handling. Treatment with ATP13A2‐ and ATP13A3‐targeted siRNA resulted in a significant reduction in mRNA levels and the simultaneous addition of these siRNAs effectively knocked down both genes (Fig. 5B). After siRNA treatment, the knockdown cells were incubated with polyamines for 1 day and subjected to polyamine quantitation. As expected, ATP13A2 knockdown resulted in reduced accumulation of exogenously added polyamines (Fig. 5C). Notably, a similar outcome was observed for ATP13A3 knockdown, indicating possible contribution of ATP13A3 to polyamine handling. Functional confirmation will require biochemical characterization of ATP13A3 protein and directionality assays.

## Discussion

To our knowledge, this is the first study to report the functional characterization of ATP13A2 expressed in cultured human lung cells. The animal polyamine transport system was enigmatic until Heinick *et al*. [12] identified the *C. elegans* homologue of ATP13A2 in mutant screening for deficiency in polyamine intake and it has been firmly established that ATP13A2 constitutes a mammalian polyamine transport system. However, its function in lungs remains unclear. The tissue and/or organ distribution of ATP13A2 pointed to its specific role in the brain where ATP13A2 is predominantly expressed [23], as also demonstrated in this study. Among other organs, the lungs appeared to have the highest levels of endogenous ATP13A2, supporting the idea that it constitutes an active polyamine uptake system in the lung epithelium. It is still unclear which site in the lung expresses ATP13A2. Nevertheless, given the results obtained in NCI‐H441 cells, ATP13A2 was most likely expressed in lung epithelial cells. The cell line was derived from the pericardial fluid of a patient with papillary lung adenocarcinoma. H441 cells have alveolar type II and/or club cell‐like phenotypes confirmed by the production of surfactant proteins and the formation of lamellar bodies [34, 35]. Notably, in H441 cells, the expression of Type II cell markers such as surfactant protein C (SP‐C) and ABCA3 gradually increased with time in culture [17]. In other words, the gene expression profile of H441 gradually changed over time toward the late differentiated state in an alveolar type II cell. This may be associated with a time‐dependent decrease in ATP13A2 expression in H441 cells. Given the DNA‐stabilizing effects of polyamines, which are widely observed in various species [36], it is not surprising that ATP13A2 was temporarily expressed during the proliferation stage before complete cell differentiation.

As previously demonstrated in various sources and models, ATP13A2 in lung cells exhibits polyamine‐dependent ATPase activity and affects intracellular polyamine distribution, confirming that the relevance of ATP13A2 as a polyamine transporter is not brain‐specific but rather systemic. Compared to the amount of overexpressed ATP13A2 in our experiments, its effect on the amount of cellular SPD seems to be rather modest. It is possible that the amount of ATP13A2 protein exceeded the physiologically optimal level and its contribution to cellular SPD levels was saturated.

We and others speculate that ATP13A2 is potentially involved in the transport of other cationic xenobiotics [13] Considering the relatively large and negative binding pockets in the ATP13A2 structures [31, 37, 38, 39, 40], it may not be unusual that ATP13A2 and its analogous transporters (P5‐ATPases) would have a somewhat broad substrate specificity. The recently solved EM structures of SPM‐bound ATP13A2 demonstrated that the substrate binding site distinguishes polyamines based on the length of the aliphatic spacers between the amine groups in the substrates [39]. It may be intriguing to question whether other similar molecules with such structural criteria are also recognized by ATP13A2. Further functional and biochemical experiments are required to assess this possibility. This study serves as a relevant resource for information and experimental tools.

## Conclusions

This study reports the functional characterization of ATP13A2 expressed in cultured human lung cells. ATP13A2 is endogenously expressed in cultured lung epithelial cells and facilitates the cellular incorporation of polyamines.

## Conflict of interest

The authors declare no conflict of interest.

## Author contributions

YH contributed to the conceptualization, data curation, formal analysis, funding acquisition, and investigation; performed project administration; and contributed to the original draft and review and editing. KK and TK contributed to the conceptualization, data curation, formal analysis, and investigation; and participated in the writing of the original draft and review and editing. TK acquired funding and participated in the review and editing. SK and MI were responsible for data curation and contributed to the review and editing. AH contributed to data curation and validation, and participated in the review and editing. HN provided resources and supervision, managed project administration, and contributed to the review and editing. MT contributed to the conceptualization, resources, funding acquisition, investigation, and supervision; performed project administration; and contributed to the review and editing.

## Supporting information


**Data S1.** Antibody list and other supporting data including gel, membrane, and confocal images.

## Data Availability

The data that support the findings of this study are openly available in DRYAD at https://doi.org/10.5061/dryad.7m0cfxq72, dataset DOI:10.5061/dryad.7m0cfxq72.
