# Supplementary material for: ATP13A2 is involved in intracellular polyamine transport in lung epithelial cells
Source: FEBS Open Bio. 2025 Nov 18;16(4):766–77. doi: 10.1002/2211-5463.70158 (PMC13042586; doi:10.1002/2211-5463.70158)
Supplement: Supplementary file 1 — Data S1. Antibody list and other supporting data including gel, membrane, and confocal images. [file FEB4-16-766-s001.pdf]

Supplementary Data 1

List of the antibodies used in the study.

List of antibodies used in the study.

	Manufacturer	Cat. #	Dilution factor	
			Western blotting	Immunostaining
Primary antibodies				
anti-ATP13A2	Sigma-Aldrich	A9607	1000:1	200:1
anti-P4HB (PDI; protein disulfide isomerase)	abcam	ab2792	1000:1	
anti-ß-actin	Sigma-Aldrich	A5441	1000:1	
anti-Lamp1	ATLAS ANTIBODIES	AMAb91170		200:1
Secondary antibodies				
polyclonal anti-rabbit IgG (H+L) Alexa Fluor 568 conjugate	Thermo Fisher Scientific	A11011		200:1
polyclonal anti-mouse IgG (H+L) Alexa Fluor 488 conjugate	Thermo Fisher Scientific	A11029		200:1
Anti-Mouse IgG, HRP-Linked	Cytiva	NA931		200:1
Anti-Rabbit IgG, HRP-Linked	Cytiva	NA934		200:1

Supplementary Data 2

A

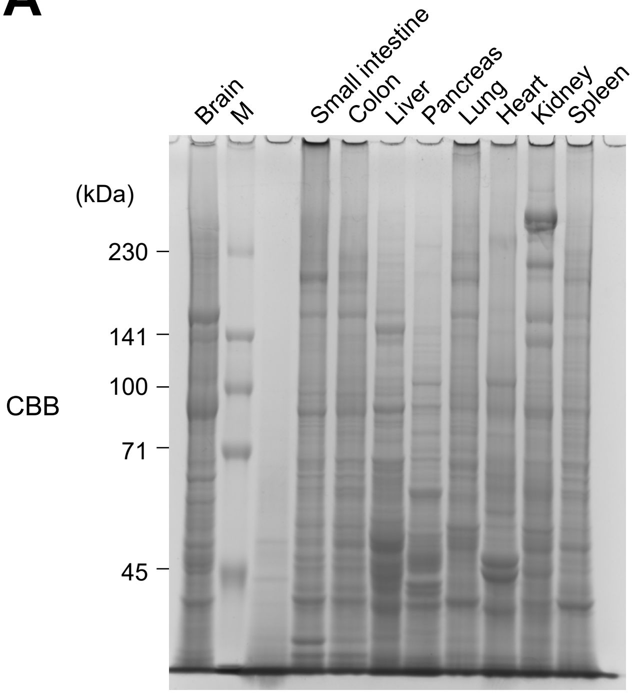

B

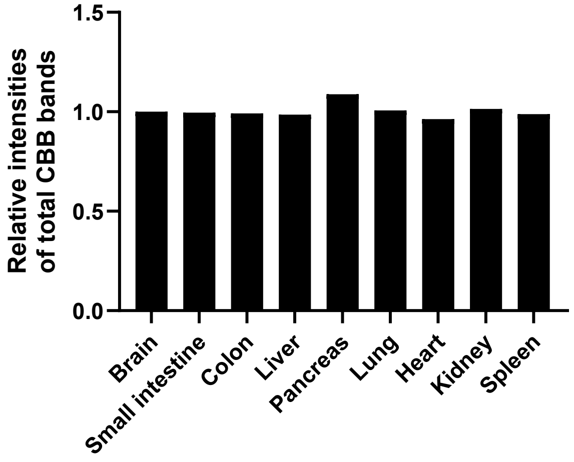

A. Coomassie Brilliant Blue-stained gel corresponding to the result shown in the main figure 1A (Western blotting detection of ATP13A2 in mouse organs). Total protein bands in each lane was quantified by densitometry and the values are shown in ***B***.

Supplementary Data 3

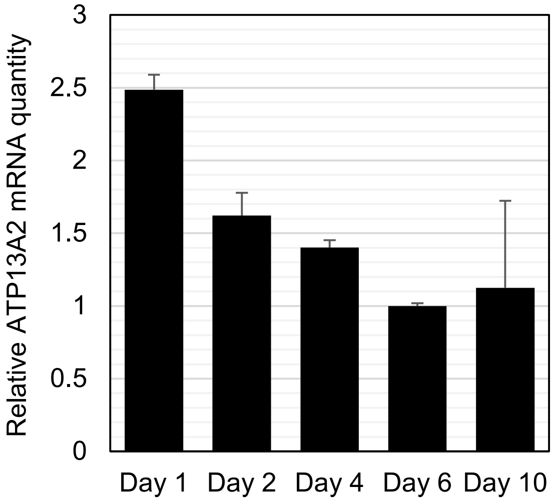

Quantification of mRNA level of ATP13A2 in H441 cells. The qPCR was performed using 2-step method and analyzed by ddCt using GAPDH as reference gene. The values are normalized to the data at Day 6. The data represent mean ± s.d. from triplicate experiments.

Supplementary Data 4

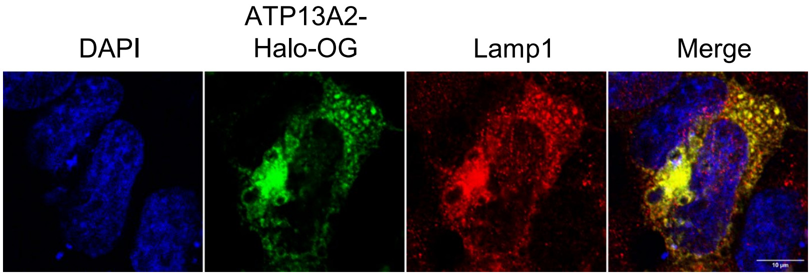

Lysosomal localization was determined by immunostaining using anti-Lamp1 antibody. The recombinant ATP13A2 was labeled with Oregon green Halo ligand (ATP13A2-Halo-OG). Scale bar, 10 μm.
